# Direct reprogramming of mouse fibroblasts into neural cells via *Porphyra yezoensis* polysaccharide based high efficient gene co-delivery

**DOI:** 10.1186/s12951-017-0317-y

**Published:** 2017-11-14

**Authors:** Qingtong Yu, Jingjing Chen, Wenwen Deng, Xia Cao, Yan Wang, Jie Zhou, Wenqian Xu, Pan Du, Qiang Wang, Jiangnan Yu, Ximing Xu

**Affiliations:** Department of Pharmaceutics, School of Pharmacy, and Center for Drug/Gene Delivery and Tissue Engineering, Jiangsu University, Zhenjiang, 212001 People’s Republic of China

**Keywords:** *Porphyra yezoensis*, Cationized polysaccharide, Gene co-delivery, nanoparticles, Nanoparticles, Neural trans-differentiation

## Abstract

**Background:**

The cell source for transplantation therapy is always a prerequisite question to be solved in clinical applications. Neural cells are considered non-regenerable, which highly restrict their application in the treatment for nerve injury. Therefore, neural trans-differentiation based on gene transfection provides a new solution to this issue. Compared to viral strategy, non-viral gene delivery systems are considered as a more promising way to achieve this aim. This study centers on a novel application of *Porphyra yezoensis* polysaccharide as a non-viral gene carrier for the neural trans-differentiation of mouse fibroblasts.

**Results:**

Ethanediamine modified *P. yezoensis* polysaccharide (Ed-PYP) served as a gene carrier and a group of plasmids that encode Ascl1, Brn4, and Tcf3 (pABT) self-assembled into nanoparticles. Results demonstrated that Ed-PYP–pABT nanoparticles at Ed-PYP: pABT weight ratio of 40:1 was the optimal candidate for gene delivery. ELISA assay revealed the highest expression levels of NGF, BDNF and SHH at 14 days after last transfection. Immunofluorescence and western blot assays also showed robust expression of neural markers including Nestin, GFAP, β-3tubulin, NF200, GAP43 and MAP2, in induced 3T6 cells at this time point.

**Conclusion:**

Overall, these findings indicated that the *P. yezoensis* polysaccharide-based non-viral gene co-delivery system is a promising strategy for the generation of neural cells, which might facilitate the developments in the recovery of neural injuries.

**Electronic supplementary material:**

The online version of this article (10.1186/s12951-017-0317-y) contains supplementary material, which is available to authorized users.

## Background

Reprogramming of somatic cell lines is considered as a promising method for cell therapy and regenerative medicine. Currently, a great deal of somatic cells such as hepatocytes [1, 2], myocardial cells [3] and neural cells [4] has been obtained through reprogramming methods. Among them, neural trans-differentiation may provide a new way for recovery and regeneration of neural system injury. In some previous studies, gene-based cellular reprogramming became a mainstream in multiple cell lines. A gene combination (Ascl1, Brn2, Myth1l), selected from 19 candidates, successfully convert mouse fibroblasts to functional neural cells through lentiviral infection [5]. Meanwhile, reprogramming factors and small chemical compounds have been also employed in trans-differentiation. MITF, SOX10 and PAX3, which were introduced in induced medium, successfully reprogrammed both mouse and human fibroblasts into melanocytes [6]. A combination of nine chemical compounds (LDN193189, SB431542, TTNPB, Tzv, CHIR99021, VPA, DAPT, SAG, Purmo) could convert astroglia into functional neural cells [7]. However, in considering of the duration, cost and passage stability, gene transfer is still the primary choice for cellular trans-differentiation research. Successful gene transfer relies on efficient gene delivery, followed by effective gene expression in target cells [6]. So far, there are two types of gene vectors, namely, viral vectors and non-viral vectors. Viral gene vectors, possessing high transfection efficiency, have been widely used for gene delivery; however, the clinical application of viral vectors is hampered by serious drawbacks such as abnormal gene expression [8–10], immune responses [11–13], limitations with respect to scale-up procedures, and inflammation [14].

To overcome these obstacles, non-viral gene vectors have been extensively investigated due to potential merits such as low toxicity, ease of synthesis, stability, and non-immunogenicity, among other factors [15–18]. Currently, cationic lipids and cationic polymers such as polyethylenimine (PEI) [19], poly-l-lysine and its derivatives [20, 21], chitosan and its derivatives [22, 23], have been investigated. Polyethylenimine (PEI), frequently utilized as a non-viral vector for its “proton sponge effect” in lysosomes [24], presented high cytotoxicity during gene transfer [25, 26], and thus needs modification prior to its use in gene therapy. However, some drawbacks like cytotoxicity and low transfection efficiency still remain [27]. Chitosan is broadly acknowledged as one of the most promising cationic gene vectors. Nevertheless, the disadvantages such as poor water solubility at physiological pH and low gene transfer efficiency, need to be solved before the practical application [28]. Recently, natural polysaccharides have shown great potential for developing as effective gene vectors after appropriate cationization due to their improved water solubility and low toxicity [29, 30]. *Porphyra yezoensis*, mainly cultivated in China, Japan, and Korean, is an important alga that is mainly processed into juice and sauce [31]. *P. yezoensis* is abundant in polysaccharides (20–40%) [32] and has demonstrated biological functions such as anti-tumor, anti-oxidation, and anti-inflammation [33–36]. Its high polysaccharide content makes it be a promising source for developing non-viral cationized polysaccharide-based gene vectors. However, it is little studied as a functional biomaterial for non-viral gene delivery.

In this work, we developed a new non-viral gene vector based on ethylenediamine-modified *P. yezoensis* polysaccharide (Ed-PYP) for co-delivery of a group of plasmids(Ascl1, Brn4, Tcf3)to a cell line of mouse embryo fibroblasts (3T6). The Ed-PYP could effectively combine with the negatively-charged plasmid DNA through electrostatic interaction and release the plasmid via the “proton sponge effect”. Moreover, instrumental characterization and cellular uptake inhibition test showed that the Ed-PYP enjoyed excellent biocompatibility, low cytotoxicity and high transfer efficiency. Importantly, the Ed-PYP/pABT nanoparticles could successfully convert mouse fibroblasts to neural cells.

Taken together, this newly developed cationized *P. yezoensis* polysaccharide was expected to be an ideal non-viral gene carrier in gene delivery system and provide a new way for neural cell generation, which might be applied to the regeneration of neural system after injury.

## Methods

### Regents and materials

Dulbecco’s modified Eagle’s medium (DMEM), penicillin–streptomycin, trypsin and fetal bovine serum (FBS) were purchased from Gibco (Gibco, USA). MTT (3-(4, 5-dimethylthiazol-2-yl)-2, 5-diphenyltetrazolium bromide) and Lipofectamine2000 were obtained from Invitrogen (Carlsbad, CA, USA). Plasmids involved in this study were obtained from Bioworld technology Inc (Nanjing, China). Ethylenediamine and polyethylenimine (PEI, 25 kDa) were procured from Sigma Chemical Co. (St. Louis, MO, USA). All other reagents were purchased from Sinopharm Chemical Reagent Co, Ltd. (Shanghai, China).

### Preparation of Ed-PYP

PYP was previously obtained from our library [35]. Then ethanediamine was added for cationization of PYP. In Brief, 1 g of PYP powder was dissolved in 100 ml double distilled water (DDW) and 1.4 g of potassium periodate was added, followed by agitating with magnetic stirrer in a darkroom for 72 h at room temperature. Subsequently, 20 ml ethylene glycol was added to react for another 30 min under the same condition before terminating the reaction. The reaction mixture was dialyzed against DDW for 2 days (3000–4000 MW cut-off filter) followed by lyophilization to obtain the oxidized PYP. In addition, 300 mg of oxidized PYP was dissolved in 60 ml DDW, thereafter 15 ml of 0.1 M borate-buffered solution (pH 9.0) containing 0.39 ml of ethylenediamine was added. The mixture was agitated with a magnetic stirrer for 24 h at room temperature and then 360 mg sodium borohydride was added to the mixture for another 48 h. Besides, 360 mg sodium borohydride was again added to the system with magnetic stirrer at room temperature for 24 h. Finally, the reaction mixture was dialyzed against DDW for 2 days (3000–4000 MW cut-off filter) to obtain ethylenediamine-introduced PYP (Ed-PYP) solution and then freeze-dried to yield Ed-PYP. PYP and Ed-PYP were separately characterized by Fourier-transform infrared (FT-IR) (Nicolet Avatar-370, Thermal Fisher Scientific, USA) to retrieve the structural information.

### Preparation and characterization of the Ed-PYP–pABT nanoparticles

Ed-PYP–pABT nanoparticles were prepared by coacervation, based on the electrostatic interaction of two oppositely charged compounds [36]. Briefly, 10 mg of Ed-PYP was fully dissolved in 1 ml of sterile water to yield a stock solution (10 mg/ml). Ascl1, Brn4, and Tcf3 solutions were prepared with nucleic-free water and mixed together at 1:1:1 ratio to form the final working stock named pABT (20 μg/ml). Aliquots (100 μl) of Ed-PYP and pABT were heated separately at 55 °C for 30 min. Equal volumes of these two solutions were immediately mixed together and vortexed for 30 s and then incubated at room temperature for 30 min to obtain Ed-PYP–pABT nanoparticles.

### Electrophoresis of Ed-PYP–pABT nanoparticles

The plasmid DNA retardation effect of the Ed-PYP–pABT nanoparticles was analyzed through 1% agarose gel electrophoresis. Different Ed-PYP: pABT weight ratios (10:1, 20:1, 40:1, 80:1, 150:1 and 300:1) of Ed-PYP–pABT nanoparticles were prepared. The Ed-PYP–pABT nanoparticles solution (5 μl) was added to 1 μl of loading buffer (0.1% sodium dodecyl sulfate, 5% glycerol, and 0.005% bromophenol blue) and applied to a 1% agarose gel in a Tris/borate/EDTA (TBE) buffer solution (pH 8.0) containing 0.1 mg/ml ethidium bromide. Meanwhile, equal volumes of naked pAscl1, pBrn4 and pTcf3 solutions prepared by nucleic-free water were employed as negative controls. Electrophoresis of Ed-PYP–pABT nanoparticles was performed in TBE solution at 80 V for 90 min. The gel was imaged with an ultra violet transilluminiator (Gel Doc 2000; Bio-Rad Laboratories Inc, Hercules, CA, USA).

### Cytotoxicity assay

The cytotoxicity of Ed-PYP–pABT nanoparticles was evaluated in vitro by MTT assay. In brief, 3T6 cells were seeded in a 96-well culture plate at an initial density of 5 × 10^4^ cells/well with serum-free medium (DMEM) and incubated at 37 °C until the cell confluence reached 80% (5% CO_2_). After 12 h of serum-free incubation, Ed-PYP/pABT nanoparticles (Ed-PYP: pABT weight ratios of 20:1, 40:1, 80:1, 150:1, 300:1, and 400:1) were transferred, and the Lipofectamine2000-pABT and PEI–pABT groups were used as positive controls according to the manufacturer’s protocol under the same conditions. Untreated cells were set up as 100% viability. The relative cell viability (%) was calculated by the formula:

$${\text{cell viability }}\left( \% \right) = \left[ {\text{Abs}} \right]_{\text{sample}} /\left[ {\text{Abs}} \right]_{\text{control}} \times 100.$$

### Zeta potential and nanoparticle-size distribution

The surface charge and average particle size was measured at 25 °C using a NanoBrook 90 Plus PALS (Brookhaven Instruments, USA).

### Transmission electron microscopy

The shape and size of the Ed-PYP–pABT nanoparticle was observed by transmission electron microscopy (TEM) (JEM2100, JEOL, Tokyo, Japan). Briefly, 1 μl of nanoparticle suspension was applied to a copper screen and air-dried. The air-dried samples were then observed directly under TEM.

### Cytophage inhibition assay

YOYO-1 is a new type of double-stranded DNA (dsDNA) fluorescent dye for tracking early intake and cell membranes, with high affinity to double-stranded DNA and a remarkable increase in fluorescence intensity after combining with DNA. Additionally, YOYO-1 alone does not have cell membrane permeability. In this work, nine different inhibitors were selected to investigate the mechanism of the Ed-PYP–pABT nanoparticles transportation; CPZ, glucose, genistein and DMA were used to investigate the intake mechanism of Ed-PYP–pABT nanoparticles. NH_4_Cl, cytochalasin D, colchicine, acrylamide and SOV were used to investigate the intracellular trafficking mechanism of ED-PYP–pABT nanoparticles. NH_4_Cl was dissolved in sterile purified water and filtered through a 0.22 µm filter and then diluted by serum-free medium (all inhibitors were diluted by serum-free media in this work) at a final concentration of 20 mM, respectively. Cytochalasin D, dissolved in dimethyl sulfoxide (DMSO), was employed at final concentration of 6 µM. Colchicine was prepared with DMSO at final concentration of 20 µg/ml. Acrylamide was dissolved in 0.5 M DMSO to obtain stock solution at final concentrations of 5 mM. CPZ and DMA, dissolved in sterile purified water, were used at final concentration of 20 µM. SOV and Genistein were dissolved in sterile purified water to form working stocks at final concentration of 10, 200 µM in respective. Glucose was dissolved in sterile purified water and then filtered through a 0.22 µm filter to obtain Glucose solution at a final concentration of 0.45 M. 3T6 cells were seeded on 96-well culture plates at an initial density of 5 × 10^4^ cells/well and incubated for 24 h in the same condition for cell culture. Cells were then treated with/without the inhibitors as described previously. After 30 min of incubation, Ed-PYP–pABT nanoparticles (40:1), labeled with YOYO-1, were added to freshly prepared 3T6 cells. After incubating at 37 °C in 5% CO_2_ for 2 h, the serum-free media was washed twice with Dulbecco’s phosphate buffered saline (DPBS) and further incubated in 500 µl of DMEM medium supplemented with 10% FBS. After 48 h incubation, the samples were fixed with 4% paraformaldehyde at 4 °C for 4 h followed by stained with Hoechst 33342 (7.5 μg/ml) for 10 min at room temperature. After washed with PBS, the samples were imaged with Axio Observer A1m (Zeise, Germany).

### Gene transfection

The 3T6 cells were seeded in 24-well plates at an initial density of 5 × 10^4^ cells/well. To identify the gene transfection efficiency, the plasmid encoding Ascl1, Brn4 and Tcf3 were mixed at an equal concentration and then co-transferred to 3T6 cells. Based on the cytotoxicity assay, electrophoresis and instrumental characterization of Ed-PYP–pABT nanoparticles, three groups of Ed-PYP–pABT nanoparticles at various Ed-PYP/pABT weight ratios (20:1, 40:1, and 80:1) were employed in this assay. Twenty-four hours after seeding, the medium was subsequently replaced with 0.5 ml of serum-free DMEM/F12 at 12 h before transfection. After removal of the medium, 0.5 ml of prepared nanoparticle suspensions (pABT 0.8 μg/well) diluted with serum-free medium were added to each well. At 48 h after transfection, the transfected 3T6 cells were scraped for western blot analysis. Meanwhile, EGFP transfection was based on the same protocol followed by microscopic examination.

### Western blot analysis

The scraped 3T6 cells were lysed in radioimmunoprecipitation assay lysis buffer with a protease-inhibitor cocktail (Merck Millipore, Billerica, MA, USA) in an ice bath for 30 min washed with PBS and sonicated for 10 s. The cell lysates were harvested from the supernatant after centrifugation at 12,000*g* for 5 min. The protein samples were mixed with an equal volume of loading buffer containing 5% β-ME and then heat denatured at 100 °C for 10 min. The volumes of loaded samples were determined according to the immunoblotting result of β-actin. Naked pABT transfected group, pABT plus Lipofectamine2000 and pABT plus PEI—transfected groups were applied as negative and positive controls in respective. All protein samples were loaded onto 12% sodium dodecyl sulfate–polyacrylamide gels, separated electrophoretically, and transferred onto polyvinylidene difluoride membranes (Merck Millipore, Billerica, MA, USA) by electrophoresis. The membranes were sequentially hybridized with primary antibody and followed with a horseradish peroxidase-conjugated secondary antibody. All the antibodies were purchased from Abcam. Thereafter, the protein bands were visualized on the Clinx Science Instruments (Shanghai) using the enhanced chemiluminescence detection system. A gel-digitizing software, ImageJ was used to quantify the relative intensity.

### Neuron conversion

The 3T6 cells were seeded in 24-well plates at an initial density of 5 × 10^4^ cells/well. In accordance with our previous study, Ed-PYP–pABT nanoparticles at a weight ratio of 40:1 were selected to transfer 3T6 cells in the following study. Here, we employed three plasmids encoding Ascl1, Brn4, and Tcf3 and mixed them up in 1:1:1 ratio and co-delivered them to 3T6 cells by Ed-PYP. After four times of transfection (once a day), DMEM/F12 was replaced with neural inducing medium (DMEM/F12 medium containing 1% B27, 2 mM and l-glutamine, 20 ng/ml FGF, 20 ng/ml EGF and 2 μg/ml heparin. We separately collected supernatant at 7, 14 and 21 days after last transfection and carried out ELISA test to determine the expression of neural factors (NGF, BDNF, Shh). In addition, immunofluorescence test and western blot were also carried out to identify the expression of neural related factors, which were Nestin, GFAP, β-3tubulin, NF200, GFAP43 and MAP2.

Nissl bodies are a series of basophilic materials in neural cells. They are the main site of protein synthesis, which directly related to the function of neural cells. Toluidine staining was carried out to identify nissl bodies in induced 3T6 cells. In addition, flow cytometry was carried out to evaluate neural conversion efficiency. The experimental process can be found in Additional file 1. Anti-neural nuclear antibody labeled Alexa-Flour 488 (diluted with PBS at 1: 100 ratio) was used to stain induced 3T6 cells. Untreated 3T6 cells and neural cells prepared from our lab were used as negative control and positive control in respective. The preparation protocol can be found in Additional file 1. The protocol was based on the manufacture’s instruction (Transcription Factor Staining Buffer Set, Thermo Fisher Science, USA).

## Results and discussion

### Ed-PYP characterization

Fourier-transform infrared (FTIR) analysis was carried out to characterize the Ed-PYP in triplicates. As Additional file 1: Figure S1 showed, the specific absorption peaks were observed at 3443.08, 2893.67, 1652.71, and 1467.01 cm^−1^. The peak at 3443.08 cm^−1^ emerged from the stretching vibration of –NH2, the peak at 2893.67 cm^−1^ came from the stretching vibration of –CH2–, and the peak at 1652.71 cm^−1^ indicated the existence of β-NH group. These results supported that PYP was successfully cationized by ethylenediamine.

### Electrophoresis of the DNA

The gel retardation assay revealed the interaction between Ed-PYP and the plasmid DNA. The Ed-PYP–pABT was prepared at different M_Ed-PYP_/M_pABT_ weight ratios while the group of free plasmids Ascl1, Brn4 and Tcf3 was applied as negative control in this study. As Additional file 1: Figure S2 shows, when the M_Ed-PYP_/M_pABT_ ratio reached 20:1, the plasmids were completely retarded. As the weight ratio increased to 100:1, the plasmids were not only retarded completely but also migrated to the opposite direction. Specifically, the stability of the combination of Ed-PYP and plasmid DNA was highly associated with the content of the Ed-PYP in Ed-PYP–pABT nanoparticles. A higher Ed-PYP: pABT weight ratio resulted in a better combination of Ed-PYP and pABT. This result was consistent with our previous study on PEI modified *P. yezoensis* polysaccharide [37].

### Cytotoxicity assay

Cytotoxicity is crucial for evaluating the safety of a gene carrier during in vitro transfection. Based on the plasmid retardation assay, the Ed-PYP–pABT nanoparticles were prepared at Ed-PYP: pABT weight ratios from 10:1 to 400:1 for the following test. Meanwhile, 3T6 cells without transfection were designated as blank and free plasmid group ABT was set as the negative control. As shown in Additional file 1: Figure S3, there was no significant difference in cell viabilities of the nanoparticles when weight ratios increased from 10:1 to 80:1. However, when the weight ratio reached 150:1, a gradual decrease was visualized in cell viability with the increase of M_Ed-PYP_: M_pABT_ ratio. Furthermore, the lowest cell viability presented at a M_Ed-PYP_: M_pABT_ ratio of 400:1, which showed no significant difference with Lipofectamine2000, but was significantly greater than that of PEI (25 kDa) at two different folds of dilutions (*P < 0.5, Student’s t-test).

PEI might induce the overexpression of caveolin-1, a molecular that partially determine the transfection efficiency and cytotoxicity of PEI [38]. So the concentration of PEI in gene delivery system should be strictly controlled. From our experiment, Ed-PYP seemed more stable and exhibited lower cytotoxicity than Lipofectamine 2000 and PEI groups, which might illustrate that Ed-PYP, could be developed into a promising non-viral gene carrier for 3T6 cell transfection.

### Zeta-potential measurement

Compared to PYP without modification, ethylenediamine modified PYP, as shown in Additional file 1: Figure S4A displayed a positive charge value of (30.52 ± 1.26) mV. Meanwhile, the zeta potential of the nanoparticles, which were prepared at three different Ed-PYP/pABT weight ratios (20:1, 40:1 and 80:1) changed from (12.32 ± 1.02) mV to an increased positive value of (26.2 ± 1.28) mV. The zeta potential measurement demonstrated that an increase in the amount of Ed-PYP led to an increased positive charge density, which provided solid evidence for the successful cationization of PYP.

### Morphology and particle size of Ed-PYP–pABT nanoparticles

As shown in Additional file 1: Figure S4B, the smallest diameter of Ed-PYP–pABT nanoparticles appeared when the Ed-PYP: pABT weight ratio reached 40:1. This is probably because the interaction between Ed-PYP and pABT. Ed-PYP–pABT nanoparticles with a lower Ed-PYP: pABT weight ratio, which exhibited a lower z potential, could result in a weak complexation with the plasmid and thus enlarged the nanoparticles, and in contrary, a higher (> 40:1) weight ratio usually led to higher zeta potential which could cause an excessive combination of more plasmids to Ed-PYP and further trigger an increase in size of nanoparticles. All the results indicated that an Ed-PYP: pABT weight ratio of 40:1 might be optimal for gene transfection.

TEM showed the morphology and particle size of nanoparticles at the Ed-PYP: pABT weight ratio of 40:1. As shown in Additional file 1: Figure S4C, most of the nanoparticles fell within a narrow range of 150–200 nm, which agreed with particles size range (195 ± 6.46) nm by DLS, as shown in Additional file 1: Figure S4D. The Ed-PYP–pABT nanoparticles prepared at the Ed-PYP: pABT weight ratio of 40:1 exhibited a uniform sphere shape, which might contribute to its conduction to cell endocytosis during transfection [39, 40].

### Transfection efficiency

**Fig. 1:**
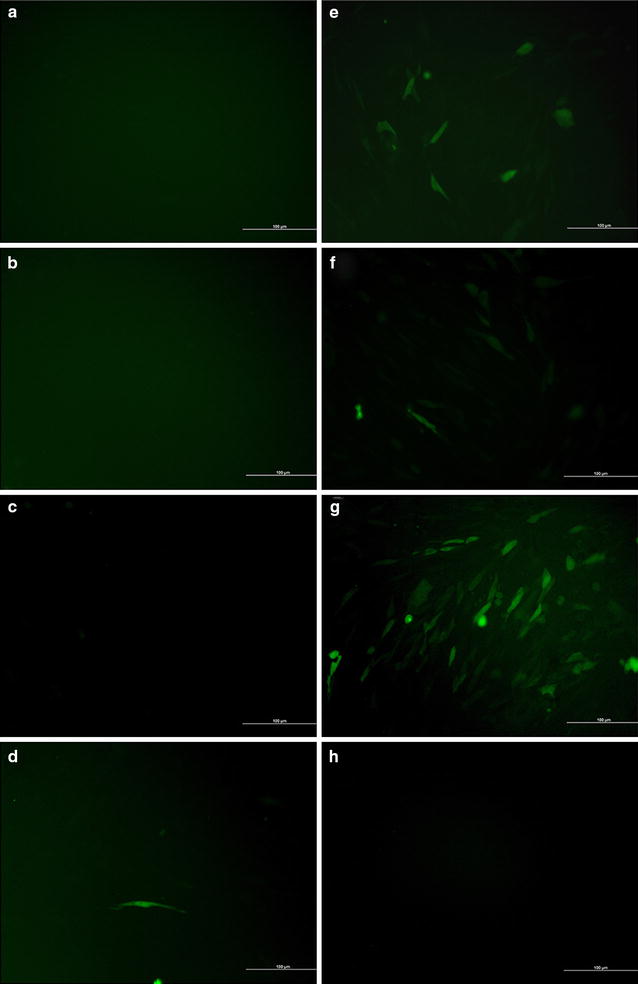
Observation of 3T6 cells transfected with plasmid EGFP (pEGFP). **a** Untreated 3T6 cells; **b** Ed-PYP treated 3T6 cells; **c** free pEGFP; **d** PEI–pEGFP; **e** lipofectamine 2000-pEGFP; **f**–**h** Ed-PYP–pEGFP nanoparticles prepared at 20:1, 40:1, and 80:1, respectively

**Fig. 2:**
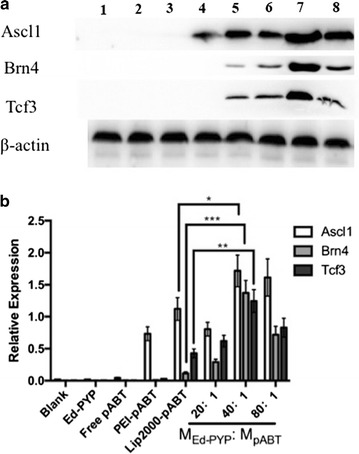
Protein-expression measurement. **a** Western blotting indicated the expression of the Ascl1, Brn4, and Tcf3 protein, using β-actin protein as the control. Lane 1, untreated 3T6 cells; lane 2, Ed-PYP treated 3T6 cells; lane 3, free plasmid group Ascl1, Brn4, Tcf3; lane 4, PEI-pABT; lane 5, Lipofectamine 2000 (Lip2000)-pABT; lane 6–8, 3T6 cells transfected with Ed-PYP–pABT nanoparticles prepared at 20:1, 40:1 and 80:1, respectively. **b** A quantitative analysis of the relative expression levels of Ascl1, Brn4, and Tcf3 (mean ± standard deviation of measurements from three replicates). *P < 0.05; **P < 0.01; ***P < 0.001; Student’s t-test

To investigate the gene transfer efficiency, we first transferred EGFP plasmid into 3T6 cells via Ed-PYP. From Fig. 1h, the Ed-PYP–pEGFP nanoparticles at the Ed-PYP: pEGFP weight ratio of 40:1 possessed the greatest expression level of GFP, which was consistence with previous characterization test. Moreover, as indicated in Fig. 2, compared to other groups, Ascl1, Brn4 and Tcf3 were all significantly up regulated after transfection with Ed-PYP–pABT nanoparticles at this weight ratio (*P* < 0.0001, Student’s *t*-test). This result indicated that Ed-PYP–pABT could be developed into a potential gene carrier for gene delivery.

### Mechanisms of cellular uptaking and intracellular trafficking

**Fig. 3:**
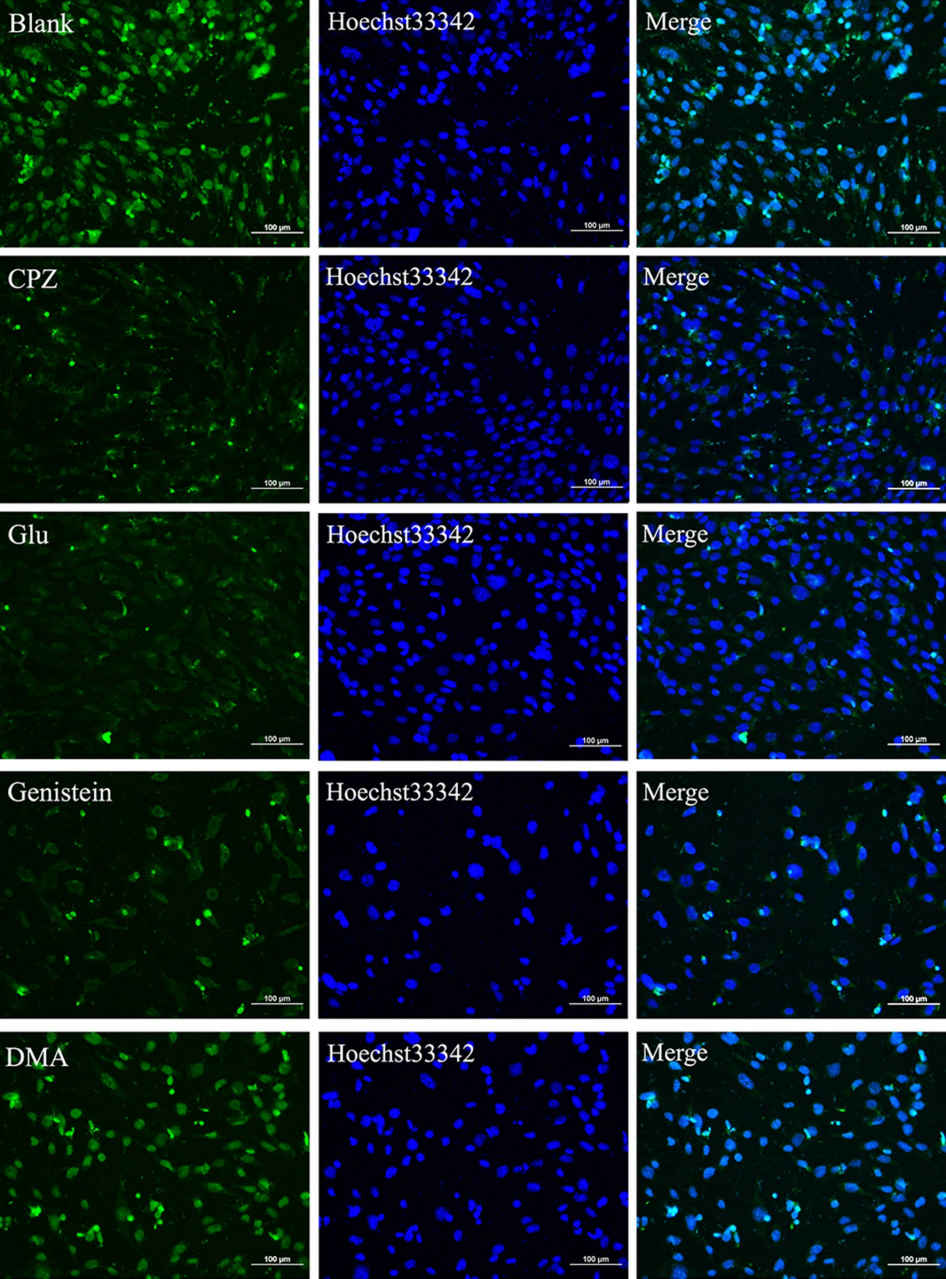
The effects of various inhibitors on the cellular uptake of Ed-PYP/pABT nanoparticles. Laser scanning confocal microscopy images of cells treated with four inhibitors (CPZ, Glucose(Glu), Genistein, and DMA) before the transfection with Ed-PYP/pABT nanoparticles (at excitation wavelengths of 405, 488 and 514 nm for blue, green and red fluorescence, respectively). Scale bars = 100 μm

We employed three types of endocytosis (clathrin-mediated endocytosis, caveolin-mediated endocytosis, and macropinocytosis) inhibitors to investigate the pathways of cellular uptake of Ed-PYP–pABT nanoparticles. The concentrations of the inhibitors were based on previous studies [41–45]. Among the three types of endocytosis inhibitors, Chlorpromazine (CPZ) and glucose were used as clathrin-mediated endocytosis inhibitors; genistein was utilized as the inhibitor of caveolin-mediated endocytosis. In addition, 5-(N,N-Dimethyl) amiloride hydrochloride (DMA) was applied for inhibiting macropinocytosis. As shown in Fig. 3, compared to the blank without any inhibitors involved, CPZ, glucose and genistein displayed remarkable inhibition effect on the cellular uptake of Ed-PYP–pABT with various degrees, among which CPZ and glucose showed stronger inhibition than genistein. Meanwhile, DMA revealed no significant inhibition. Eukaryotic cells have a variety of transfer pathways to exchange material and fulfill cellular functions. Small molecules such as inorganic ions and glucose are primarily taken up through passive diffusion or active transport, while the uptake of macromolecular or granular material by eukaryotic cells requires various endocytic pathways. Endocytosis can generally be divided into two types, namely, phagocytosis and pinocytosis. Solid particles can be taken up by phagocytosis, whereas fluid and solution uptake could be triggered by endocytosis. Phagocytosis is limited to a number of specific cells, such as macrophages and neutrophils. Pinocytosis is subdivided into several types such as clathrin-mediated endocytosis, caveolin-mediated endocytosis, clathrin-independent endocytosis, caveolin-independent endocytosis and macropinocytosis [46–48], among which clathrin-independent and caveolin-independent endocytosis mainly function in cell signaling aspects [49]. Therefore, our present work primarily focused on clathrin-mediated endocytosis, caveolin-mediated endocytosis, and macropinocytosis, which might play important roles in Ed-PYP/pABT nanoparticles cellular uptake. Compared to the blank control without inhibitors, CPZ and glucose significantly inhibited the cellular uptake of YOYO-1 labeled Ed-PYP–pABT nanoparticles, which demonstrated that clathrin-mediated endocytosis was one of the pathways for the cellular uptake of the complex. However, CPZ and glucose did not completely inhibit the cellular uptake of the Ed-PYP–pABT nanoparticles, which suggests that endocytosis of the Ed-PYP–pABT nanoparticles might involve multiple pathways. Genistein could also inhibit the cellular uptake of the Ed-PYP–pABT nanoparticles with lower inhibition efficiency than that of CPZ and glucose. Meanwhile, DMA displayed almost no inhibition to Ed-PYP–pABT nanoparticles, which illustrated that macropinocytosis might not be involved in the cellular uptake of Ed-PYP–pABT nanoparticles. In summary, we inferred that clathrin-mediated endocytosis was the dominant method of cellular uptake of the Ed-PYP–pABT nanoparticles while caveolin-mediated endocytosis was a complementary pathway.

**Fig. 4:**
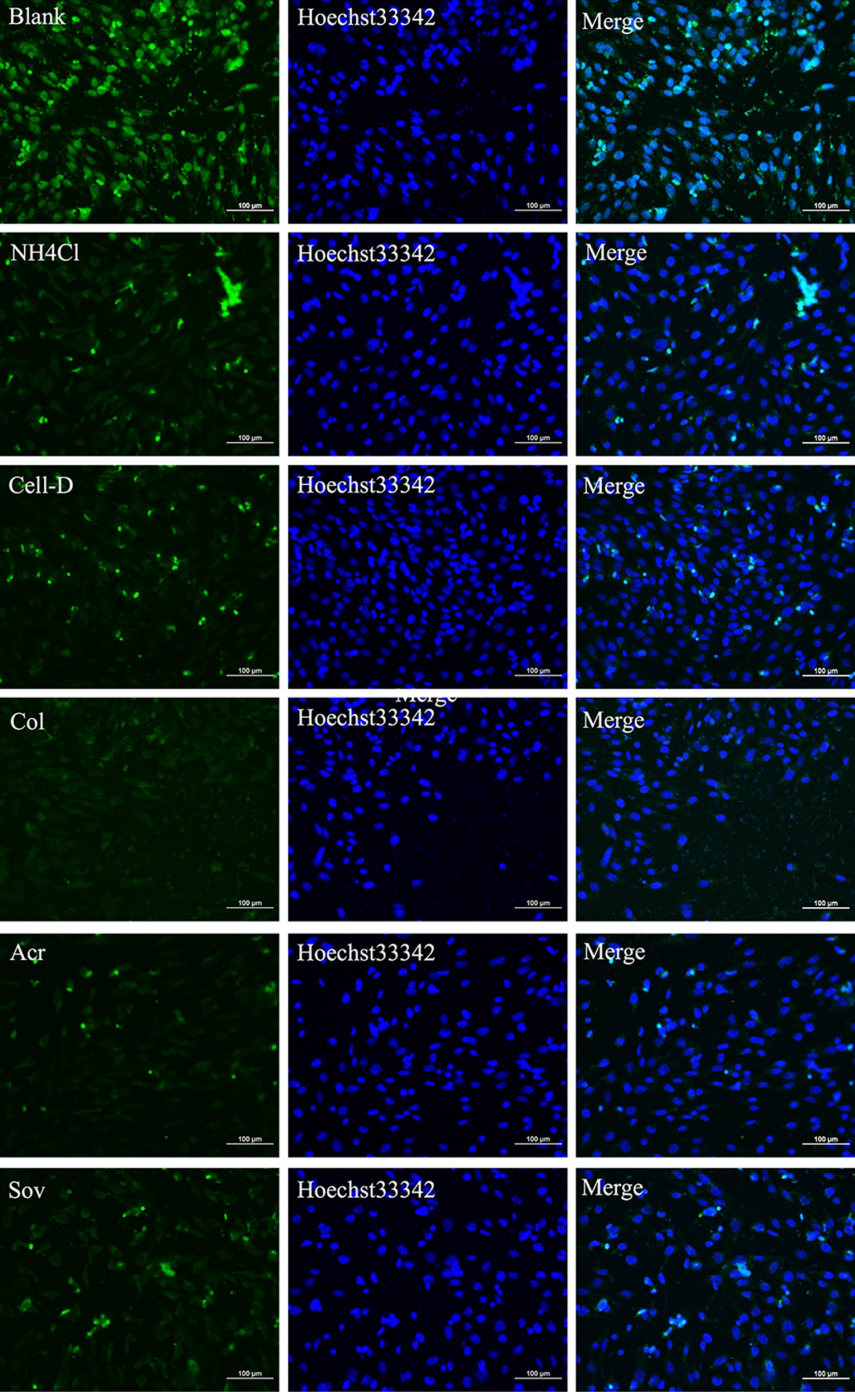
The effects of various inhibitors on the intracellular trafficking of Ed-PYP/pABT nanoparticles. Laser scanning confocal microscopy images of cells treated with five inhibitors (NH_4_Cl, Cell-D, Colchicine(Col), Acr, and SOV) before the transfection with Ed-PYP/pABT nanoparticles (at excitation wavelengths of 405, 488 and 514 nm for blue, green and red fluorescence, respectively). Scale bars = 100 μm

In intracellular trafficking assay, we employed four types of inhibitors, NH_4_Cl, Cytochalasin D, Colchicine, Acrylamide, and Sodium orthovanadate, which were respectively corresponded to endosome–lysosome system, cytoskeleton, intermediate filament and motor protein. In Fig. 4, compared to the untreated group, all the tested groups exhibited significant inhibition effects. This result indicated that the Ed-PYP–pABT nanoparticles enjoyed multiple pathways during intracellular transportation.

The uptake of nanoparticle was effected by many factors such as particle shape and size, surface modification and materials. Macropinocytosis is unusually defined as trapping of large fluid phases in the form of irregular endocytic vesicles (up to 5 μm in diameter). Interestingly, it was reported that cellular uptake quantity of spherical nanoparticles was five times compared to that of rod-like nanoparticles through endocytosis. It was believed that it requires more time to encapsulate rod-like nanoparticles by cells [50]. This might explain that Ed-PYP–pABT nanoparticles were more likely be uptaken by MEF cells. Currently, the relationship between nanoparticles size and endocytotic rate as well as endocytotic quantities still has not been elucidated. The efficiency might be related to the materials, target cell lines, et al. Chithrani et al. studied the effect of gold nanoparticles and with uniform sizes ranging from 14 to 74 nm. The result showed that 50 nm group can be most effectively adsorbed by cell membranes, which indicated that there was an optimized nano-size particles for cellular uptake [51]. Qaddoumi et al. compared respective endocytosis of PLGA nanoparticles with various size (100, 800 nm, 10 μm) in the rabbit eye conjunctival epithelial cell layer and they found the 100 nm group can be internalized via endocytosis [52]. Meanwhile, Dawson et al. reported the internalization of germ-modified PLGA nanoparticles with different sizes (155, 200, 375, 600 nm respectively) by HEp22B (human Caucasian larynx carcinoma). The endocytosis of 375 and 600 nm PLGA nanoparticles is greater than other two groups [53]. From the above discussions, it can be seen that the nanoparticle size effect needs to be studied for different nanoparticles separately. In addition, the nanoparticle size must be effectively controlled in the experiments to avoid inaccurate results.

### Neural conversion

**Fig. 5:**
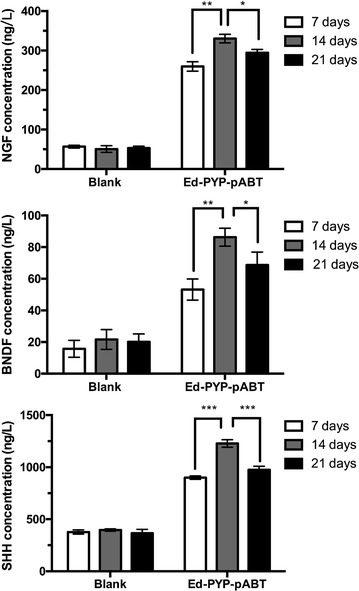
NGF, BNDF and SHH expression measured by ELISA

**Fig. 6:**
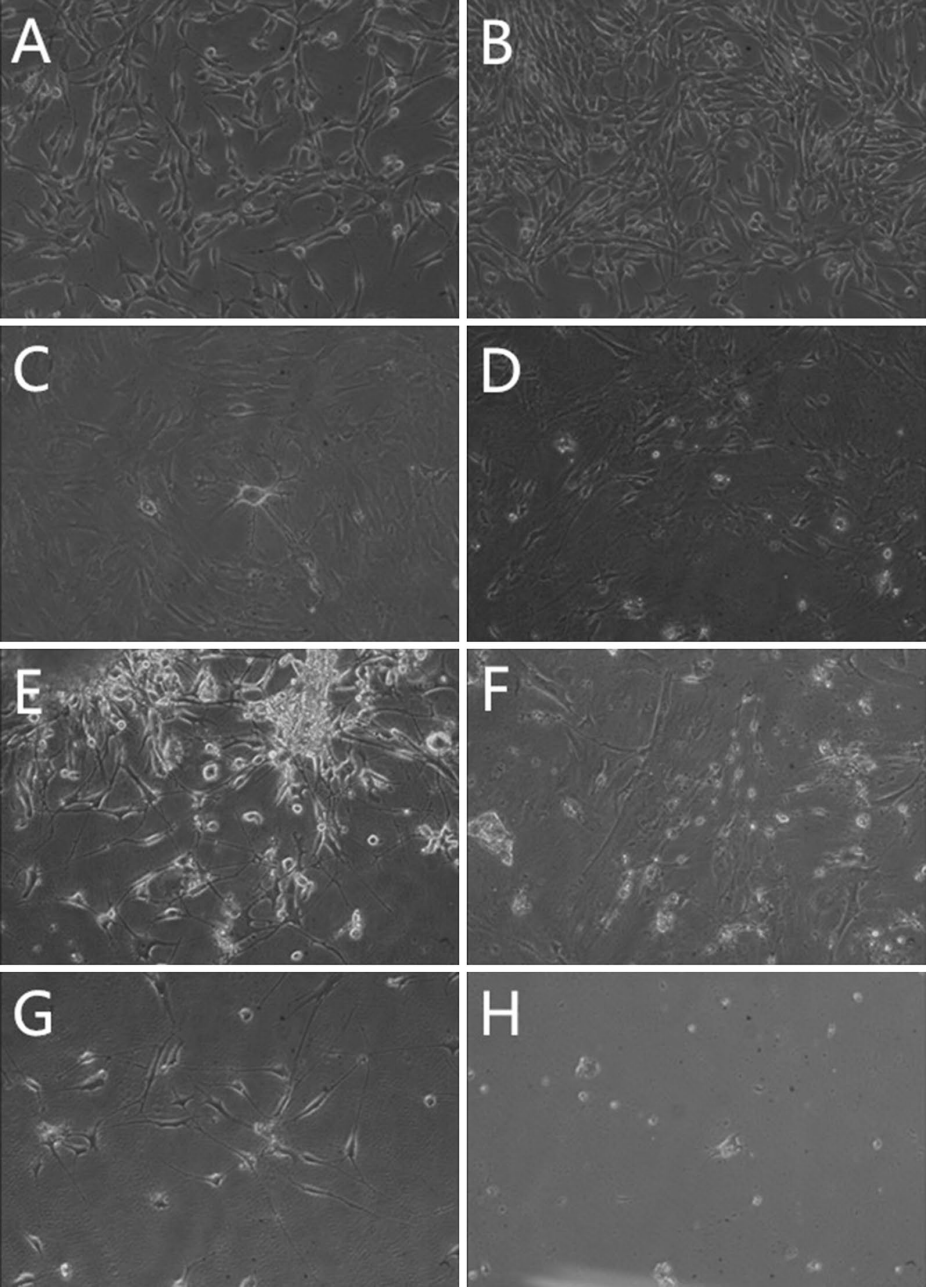
Morphological observation. **A**–**G** Induced 3T6 cells at 1, 4, 7, 10, 14, 17, and 21 days after the last transfection in respective; **H** untreated 3T6 cells at 7 days after the last transfection

**Fig. 7:**
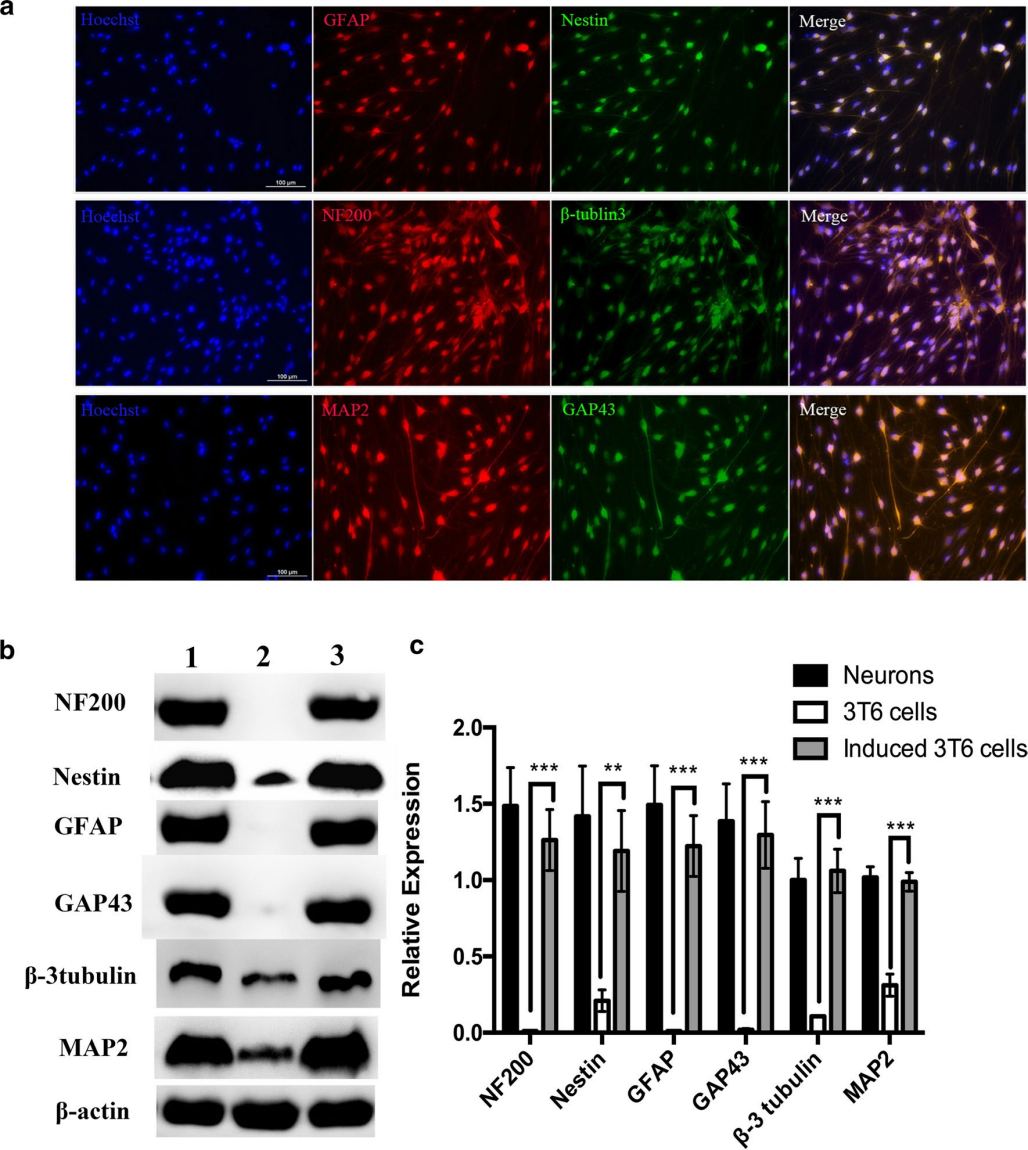
Neural conversion **a** immunofluorescence analysis of neural markers; **b** western blotting indicated the expression of neural markers; **c** a quantitative analysis of the relative expression levels of neural markers (mean ± standard deviation of measurements from three replicates). *P < 0.05; **P < 0.01; ***P < 0.001; Student’s t-test

**Fig. 8:**
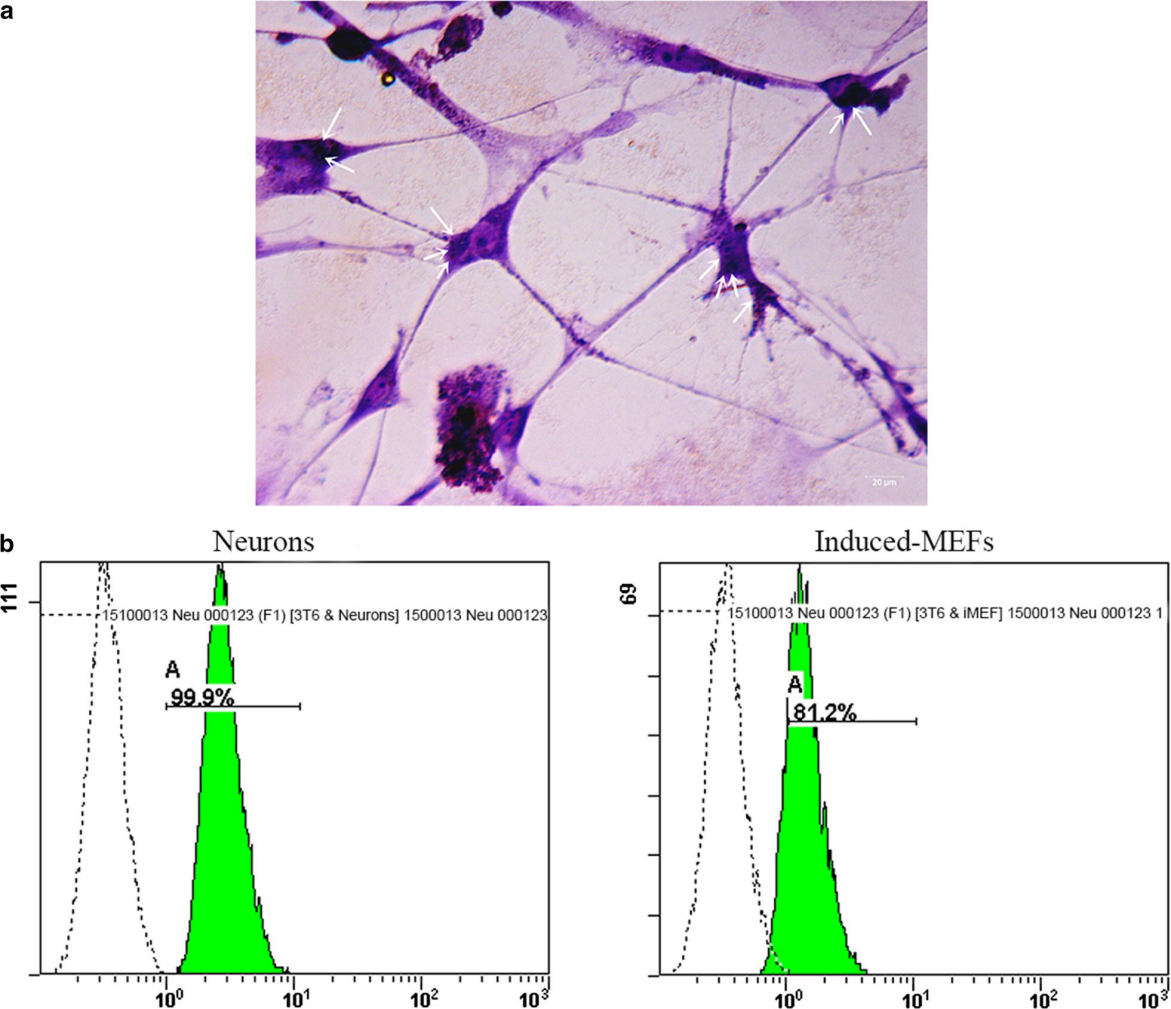
Conversion efficiency. **a** Nissl staining (showed by arrows); **b** flow cytometry analysis of induced 3T6 cells (right) and neural cells (left)

The induced 3T6 cells started to display neuron-like morphology at about 7 days after last transfection (Data not shown). To optimize the protocol of neural conversion, we separately collected the supernatant at 7, 14 and 21 days after last transfection. ELISA was carried out to determine the concentrations of the three neural related factors, which were brain-derived neurotrophic factor (BNDF), nerve growth factor (NGF) and Sonic hedgehog (SHH). From Fig. 5, at 14 days after last transfection, all the factors exhibited the highest concentration. Meanwhile, the morphology of induced 3T6 cells were observed through microscope (Fig. 6). As Fig. 6 showed, at 14 days after last transfection (Fig. 6E), nearly all the 3T6 cells possessed neural-like morphology. The cell number decreased from day 14 (Fig. 6E) to day 21 (Fig. 6G) after last transfection. Untreated 3T6 cells were set up as negative control. As we can see in Fig. 6H, obvious cell death can be found at 7 days after the medium was changed into neural induced medium. Furthermore, we carried out western blot and immunofluorescence to test some neural markers, which are GFAP, Nestin, NF200, β-tubulin3, MAP2 and GAP43 for induced 3T6 cells at this time point. As shown in Fig. 7a, immunofluorescence assay illustrated positive expression of all the neural markers involved in this test. In Fig. 4b, c, western blot indicated that the expressions of these markers in the induced 3T6 cells were significantly higher than those in untreated 3T6 cells. In addition, the induced 3T6 cells processed comparable expression levels to neural cells (positive control). Nissl staining revealed the existence of nissl bodies (Fig. 8a). Flow cytometry showed the positive rate reached 81.2% (Fig. 8b), which indicated the neural conversion efficiency is about 80%. These results indicated that we successfully convert 3T6 cells into neurons via co-delivery of Ascl1, Brn4, and Tcf3 through Ed-PYP. Yoshihiro and his colleagues [54] successfully convert human fibroblasts into Schwann cells through retrovirus encoding Sox10, Krox20 and Oct6. The conversion efficiency was evaluated through positive rate of immunofluorescence staining, which is about 43%. Sun Min Kim, etc. converted MEF cells into neural stem cells through Epstein-Barr virus (EBV) based vector and evaluated the reprogramming efficiency (47.7%) by FCM [55]. Although the objectives of these studies are different from our research work, the Ed-PYP still shows remarkable transfection efficiency as a non-viral gene vector.

Based on several candidates consist of Ascl1, Brn2, Brn4, Tcf3, and Myt1 l, we randomly combined them and set up a series of gene pools to transfect 3T6 cells. None of these gene pools could convert 3T6 cells into neurons except co-delivery of Ascl1, Brn4 and Tcf3 (data not shown). Contrary, Thomas [5] successfully converted fibroblasts into functional neurons through overexpression of Ascl1, Brn2 and Myt1l by lentivirus as gene carriers. This result was not obtained from our non-viral system. The discrepancy might be due to the different mechanisms of gene delivery through viral and non-viral transfection systems, which needs further investigation.

## Conclusions

Here we present a novel cationized polysaccharide Ed-PYP as a gene carrier for generating non-viral induced neural cells via co-delivery of plasmid group Ascl1, Brn4, and Tcf3 (pABT). The nanoparticles possessed an ultra-small size (< 200 nm), spherical shape, positive zeta potential and excellent plasmid retention when the Ed-PYP: pDNA weight ratio reached 40:1. In addition, multiple pathways of cellular uptake and intercellular trafficking successfully guaranteed gene delivery efficiency and therefore facilitated gene expression. Based on this novel gene carrier, a group of plasmids (Ascl1, Brn4, and Tcf3) elected from a series of candidate gene pools was loaded with Ed-PYP for the following neural trans-differentiation research. After four times of gene transfection, 3T6 cells were finally induced to neural cells at 14 days since the final transfection, which was proved by both western blot assay and immunofluorescence. These findings demonstrate that polysaccharide-based gene carrier would provide a new platform for safe and efficient gene delivery and therefore be applied in cellular trans-differentiation.

## Additional file

**Additional file 1: Figure S1.** Fourier-Transform infrared spectra of Porphyra yezoensis polysaccharide (PYP) and ethylenediamine modified Porphyra yezoensis polysaccharide (Ed-PYP). **Figure S2.** Electrophoretic mobility of plasmid in Ed-PYP-plasmid Ascl1, Brn4 and Tcf3 (pABT) nanoparticles at various weight ratios. Lane 1–3, free plasmids Ascl1, Brn4 and Tcf3 from left to right; Lane 4–9, Ed-PYP: pABT weight ratios of 10:1, 20:1, 40:1, 80:1, 150:1, and 300:1, respectively. **Figure S3.** Cytotoxicity assay. Bar 1, 3T6 cells treated with Ed-PYP; bar2, free plasmid group Ascl1, Brn4 and Tcf3 (pABT) (control group); bars 3–8, ethylenediamine modified *Porphyra yezoensis* polysaccharide (Ed-PYP)–pABT nanoparticles at ratios of 20:1, 40:1, 80:1, 150:1, 300:1, and 400:1 from left to right, respectively; bar 9, PEI–pABT; bars 10, Lipofectamine 2000(Lip2000)-pABT. **Figure S4.** Characterization of ethylenediamine modified *Porphyra yezoensis* polysaccharide (Ed-PYP)-pABT nanoparticles. (A) Zeta-potential results. Notes: Bar 1, naked plasmid group Ascl1, Brn4 and Tcf3 (pABT); bar 2, *Porphyra yezoensis* polysaccharide (PYP); bar 3, ethylenediamine modified *Porphyra yezoensis* polysaccharide (Ed-PYP); bars 4–6, Ed-PYP–pABT nanoparticles prepared at various Ed-PYP: pABT weight ratios-20:1, 40:1, and 80:1 from left to right, respectively (means±standard deviation of measurements from three replicates). (B) Size distribution of the nanoparticles at Ed-PYP: pABT weight ratios of 20:1, 40:1, and 80:1, respectively. (C) Transmission electron microscopy image of the nanoparticles at an Ed-PYP: pABT weight ratio of 40:1. (D) Particle-size distribution of the nanoparticles at an Ed-PYP: pABT weight ratio of 40:1.
