# Supplementary material for: Direct reprogramming of mouse fibroblasts into neural cells via Porphyra yezoensis polysaccharide based high efficient gene co-delivery
Source: J Nanobiotechnology. 2017 Nov 14;15:82. doi: 10.1186/s12951-017-0317-y (PMC5686901; doi:10.1186/s12951-017-0317-y)
Supplement: Supplementary file 1 — Additional file 1: Figure S1. Fourier-Transform infrared spectra of Porphyra yezoensis polysaccharide (PYP) and ethylenediamine modified Porphyra yezoensis polysaccharide (Ed-PYP). Figure S2. Electrophoretic mobility of plasmid in Ed-PYP-plasmid Ascl1, Brn4 and Tcf3 (pABT) nanoparticles at various weight ratios. Lane 1–3, free plasmids Ascl1, Brn4 and Tcf3 from left to right; Lane 4–9, Ed-PYP: pABT weight ratios of 10:1, 20:1, 40:1, 80:1, 150:1, and 300:1, respectively. Figure S3. Cytotoxicity assay. Bar 1, 3T6 cells treated with Ed-PYP; bar2, free plasmid group Ascl1, Brn4 and Tcf3 (pABT) (control group); bars 3–8, ethylenediamine modified Porphyra yezoensis polysaccharide (Ed-PYP)–pABT nanoparticles at ratios of 20:1, 40:1, 80:1, 150:1, 300:1, and 400:1 from left to right, respectively; bar 9, PEI–pABT; bars 10, Lipofectamine 2000(Lip2000)-pABT. Figure S4. Characterization of ethylenediamine modified Porphyra yezoensis polysaccharide (Ed-PYP)-pABT nanoparticles. (A) Zeta-potential results. Notes: Bar 1, naked plasmid group Ascl1, Brn4 and Tcf3 (pABT); bar 2, Porphyra yezoensis polysaccharide (PYP); bar 3, ethylenediamine modified Porphyra yezoensis polysaccharide (Ed-PYP); bars 4–6, Ed-PYP–pABT nanoparticles prepared at various Ed-PYP: pABT weight ratios-20:1, 40:1, and 80:1 from left to right, respectively (means±standard deviation of measurements from three replicates). (B) Size distribution of the nanoparticles at Ed-PYP: pABT weight ratios of 20:1, 40:1, and 80:1, respectively. (C) Transmission electron microscopy image of the nanoparticles at an Ed-PYP: pABT weight ratio of 40:1. (D) Particle-size distribution of the nanoparticles at an Ed-PYP: pABT weight ratio of 40:1. [file 12951_2017_317_MOESM1_ESM.docx]

Supplementary information


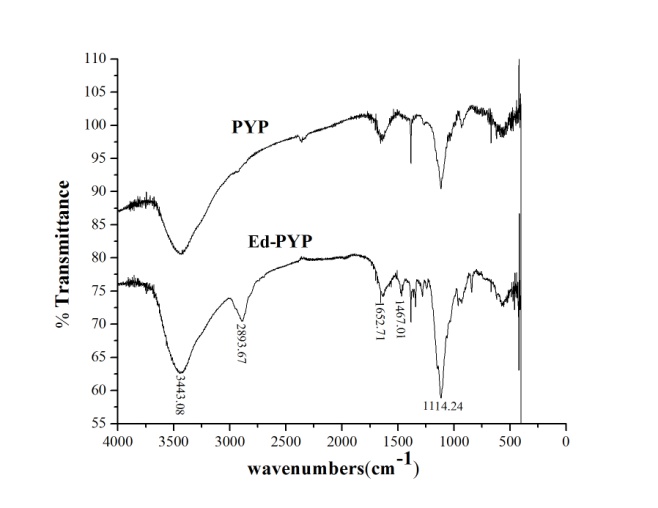


Figure 1


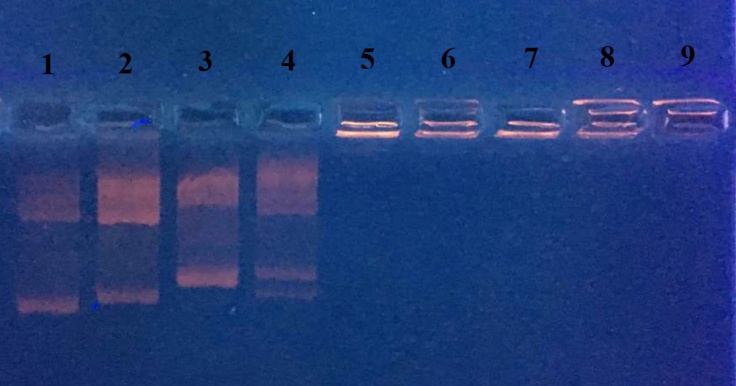


Figure 2


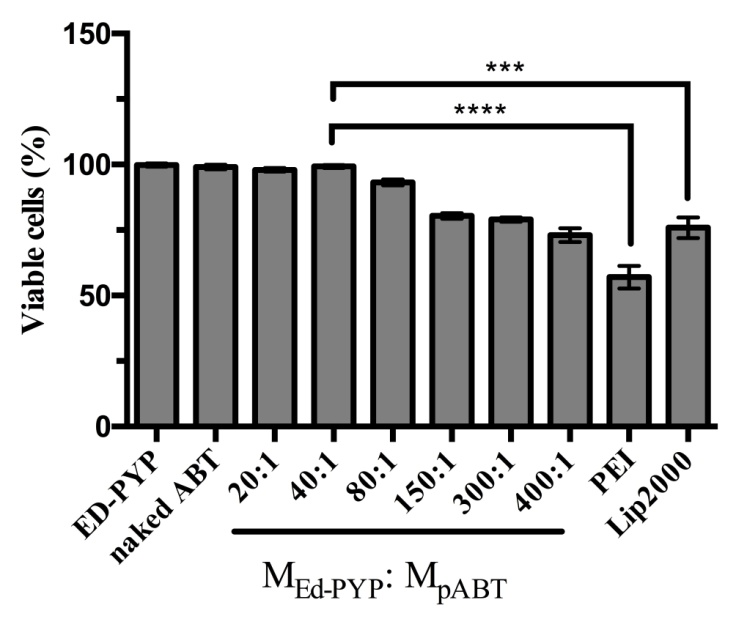


Figure 3


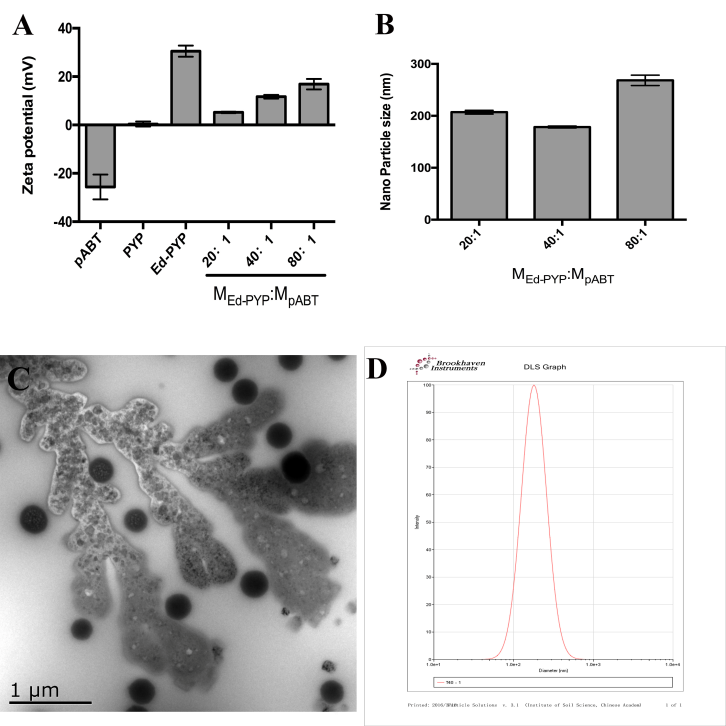


Figure 4

Nissl staining

1. Induced 3T6 cells were washed with PBS for 3 times and then fixed with 4% paraformaldehyde at room temperature for 30 min.
2. 0.5% toluidine blue was added into the plate and incubated for 40 min.
3. Cells were washed with double distilled water and 70% ethanol for 30s respectively.
4. Dehydration with absolute ethanol for 1min

Neural cells preparation

1. Select 5 neonatal mice and killed.
2. Put the bodies into iodine for 1.5 min followed by washing with 75% ethanol.
3. Scissor the scalp and take out the brain with tweezers and wash the brain with cold PBS solution.
4. Strip the meninx and scissors the brain into small pieces (about 2mm^3^ each) followed by washing them with cold PBS.
5. Centrifuge the tissue (900rpm for 5 min) and eliminate the supernatant followed by adding collagenaes type 1 and digested in 37 ℃ for 20 min.
6. Centrifuge again (900 rpm for 5 min) and eliminate the supernatant followed by adding cold PBS to resuspend the tissue.
7. Filter the suspension with cell strainer (pore diameter=57μm).
8. Centrifuge again (900 rpm for 5min) and eliminate the supernatant followed by adding serum-free medium (DMEM/F12 98ml, bFGF 20ng/ml, EGF 20ng/ml, B27 2ml, [Penicillin-Streptomycin](javascript:;) 100U/ml) to incubate.
